# Patient-pharmacist relationship dynamics: a mediation analysis of patient characteristics and reported outcomes

**DOI:** 10.1080/20523211.2024.2371409

**Published:** 2024-07-15

**Authors:** Hala Sacre, Chadia Haddad, Fouad Sakr, Jihan Safwan, Aline Hajj, Rony M. Zeenny, Marwan Akel, Pascale Salameh

**Affiliations:** a INSPECT-LB (Institut National de Santé Publique, d’Épidémiologie Clinique et de Toxicologie-Liban), Beirut, Lebanon; b School of Medicine, Lebanese American University, Byblos, Lebanon; c Research Department, Psychiatric Hospital of the Cross, Jal Eddib, Lebanon; d School of Pharmacy, Lebanese International University, Beirut, Lebanon; e Faculté de Pharmacie, Université Laval, Québec, Canada; f Oncology Division, CHU de Québec Université Laval Research Center, Québec, Canada; g Department of Pharmacy, American University of Beirut Medical Center, Beirut, Lebanon; h Faculty of Pharmacy, Lebanese University, Hadath, Lebanon; i Department of Primary Care and Population Health, University of Nicosia Medical School, Nicosia, Cyprus

**Keywords:** Patient-pharmacist relationship, Patient characteristics, Health status, Medication adherence, Quality of life, Mediation analysis

## Abstract

**Background:**

While previous research underscores the independent effect of the pharmacist-patient relationship on patient outcomes, it did not delve further into the patient-pharmacist relationship dynamics and their effects on reported outcomes. Therefore, this study aimed to assess whether patient-pharmacist relationship aspects mediate the association between patient personal and health characteristics, on the one hand, and adherence to medication and quality of life, on the other hand (QOL).

**Methods:**

An online cross-sectional study was conducted between April 11 and 27, 2023. It enrolled 865 adults from all Lebanese governorates and used validated scales to measure the various concepts.

**Results:**

The mean age was 32.52 ± 14.56 years, and 68.8% were female. Also, 79.3% reported having no chronic disease, and 57.7% indicated that getting nonprescription medications was the main reason for visiting a community pharmacy. The average routine intake of medications per day was 0.87 ± 1.78. Our key findings reveal a compelling association between worse health status and both increased medication non-adherence and reduced QOL. Sociodemographic factors were found to be correlated with QOL. Despite the considerable impact of demographic factors on patient expectations, our study challenges the expected mediation role of the pharmacist-patient relationship and counseling time on medication adherence. Nevertheless, patient expectations partially mediated the relationship between sociodemographic characteristics and QOL.

**Conclusion:**

This study sheds light on the intricate dynamics between patient characteristics, health status, medication adherence, and QOL within the context of the patient-pharmacist relationships.

## Introduction

Medication adherence plays a crucial role in determining health outcomes and overall well-being (Aremu et al., 2022; Jimmy & Jose, 2011). It is a cornerstone of successful healthcare interventions, directly affecting treatment efficacy and the prevention of adverse events (Burnier, 2006; Sakr et al., 2022a). Research has consistently shown that patient sociodemographic characteristics can significantly impact medication adherence (Sakr et al., 2022a). For instance, monthly income and financial well-being are often associated with unequal access to healthcare resources, potentially hindering patients from obtaining and adhering to prescribed medications (Kvarnström et al., 2021). Similarly, marital status and work status may influence the support system available to individuals, affecting their ability to adhere to complex medication regimens (Jin et al., 2008; Kardas et al., 2013).

Beyond medication adherence, sociodemographic factors also influence quality of life, a multidimensional construct encompassing physical, mental, and social well-being, closely linked to health outcomes (Post, 2014). Patients with compromised sociodemographic characteristics may face barriers that extend beyond medication adherence, affecting their overall quality of life (Marzo et al., 2022). Furthermore, health characteristics, such as age, health status, and chronic diseases, significantly impact both medication adherence and quality of life (Ge et al., 2019; Rolnick et al., 2013). For instance, age can influence medication adherence due to variations in cognitive abilities, physical limitations, and evolving healthcare needs across the lifespan (van de Vijver et al., 2023). Similarly, the health status of patients, especially those with chronic conditions that may necessitate complex treatment regimens, can influence their ability to adhere to prescribed medications (Chauke et al., 2022). The burden of chronic diseases, stemming from the inherent demands of daily life, introduces additional challenges that can affect both adherence and overall quality of life (Sakr et al., 2022b; Yoon et al., 2023).

The intersection of patient sociodemographic and health characteristics underscores the need for a tailored approach to healthcare delivery. In this context, pharmacists are essential contributors to bridging the gap between patients and optimal health outcomes (Eldooma et al., 2023; Sakr et al., 2023a). The relationships between pharmacists and patients and the time dedicated to counseling sessions have emerged as potential transformative factors in enhancing medication adherence and improving quality of life (Birand et al., 2019; Elnaem et al., 2020). Leveraging their expertise, pharmacists are in a unique position to address the diverse needs of patients, making a substantial contribution to their healthcare journey (Spinewine et al., 2012). A strong pharmacist-patient relationship, characterised by trust, communication, and mutual understanding, could catalyse improved medication adherence (Cernasev et al., 2021; Mu et al., 2020). Moreover, the time allocated by pharmacists to counseling sessions presents a valuable opportunity to address patient concerns, clarify uncertainties, and reinforce the importance of adhering to prescribed medications (Bou-Saba et al., 2023; Ilardo & Speciale, 2020; Sanii et al., 2016).

### Previous findings that prompted this analysis

Previous findings from the IMPHACT-LB project, a broader initiative aimed at showcasing the impact of the modern pharmacy concept on patient therapy in Lebanon, highlighted significant correlations between medication adherence and specific factors. Notably, spending more than 10 min receiving counseling from the pharmacist and age were independently linked to improved medication adherence. Conversely, factors such as visiting a pharmacy for chronic and nonprescription medications and irregular health coverage indicative of lower socioeconomic status were associated with lower adherence (Sakr et al., 2023b). Furthermore, patients who perceived pharmacists as knowledgeable medication counselors and regularly visited community pharmacies reported a better quality of life. These positive perceptions independently counteracted the negative impact of chronic diseases and multiple medications, particularly pertinent in Lebanon amidst various economic, social, and health crises (Hajj et al., 2023).

While these findings underscore the independent effect of the pharmacist-patient relationship on patient outcomes, the study did not delve further into the patient-pharmacist relationship dynamics and their specific effects on reported outcomes. Therefore, this study aimed to assess whether patient-pharmacist relationship aspects mediate the association between patient personal and health characteristics, on the one hand, and adherence to medication and quality of life, on the other hand.

## Methods

### Study design

This cross-sectional study is part of the first phase of the IMPHACT-LB project, a broader initiative that focuses on the Impact of the Modern PHArmacy Concept on Patient Therapy in Lebanon. It employed a convenience sampling technique to recruit 865 participants between April 11 and April 27, 2023. The sampling unit consisted of individuals aged 18 and older residing in Lebanon. The initial participants were recruited through social media platforms (Facebook, Instagram, and WhatsApp) using a survey instrument created on Google Forms. They were encouraged to share the survey with their networks, leading to further recruitment (snowball sampling). Although no randomisation technique was used, the sample was stratified according to region to ensure representativeness across all Lebanese governorates (Beirut, Mount Lebanon, North, South, and Beqaa).

Information about the purpose and duration of the study was provided in the questionnaire’s introduction section. Participants were also informed that their involvement was voluntary and that they might end it at any moment. They provided written informed consent before being routed to the survey and were not compensated for their participation.

### Ethical considerations

The Ethics and Research Committee at the Lebanese International University School of Pharmacy approved this protocol (2023RC-013-LIUSOP). The Helsinki Declaration’s ethical guidelines were followed in the conduct of this study.

### Sample size calculation

G-Power software, version 3.0.10, was used to determine the minimum sample size. Anticipating a squared multiple correlation of 0.05 (R2 departure from 0) related to the Omnibus test of multiple regression, the computed effect size was 0.0526. With a 5% alpha error, 80% power, and 25 predictors in the model, n = 454 was the minimal size needed. It was intended to include at least 600 participants to account for any missing data. This minimum sample size would also be sufficient to conduct a mediation involving three variables in the path analysis (Sim et al., 2022), as in the present study.

### Survey tool

The questionnaire was in Arabic, the official language in Lebanon. It consisted of two parts. The first part gathered participants’ sociodemographic characteristics (age, education level, occupation, and income). It also included questions about the main reason for visiting a pharmacy and the time pharmacists spent counseling.

The second part consisted of several validated measures.

#### The incharge financial distress/financial (IFDFW)

The InCharge Financial Distress/Financial (IFDFW) scale was used to assess financial distress and the effect of economic hardship among participants. This 8-item subjective measure has been validated in the Lebanese population (Sacre et al., 2023a). Responses were reported on a linear scale from 1 (overwhelming stress) to 10 (no stress at all), with higher scores indicating better financial well-being (Prawitz et al., 2006). In this study, the sampling adequacy and reliability measures were excellent (Kaiser-Meyer-Olkin = 0.940; Bartlett’s test of Sphericity *p*-value < 0.001; Cronbach alpha = 0.944).

#### The pharmacist-patient relationship measurement tool

The pharmacist-patient relationship was assessed using the validated pharmacist-patient relationship measurement tool (Bou Raad et al., 2017) and questions inspired by previous studies. This validated tool measured the patients’ level of expectations (Patient Expectation Index) from a visit to a community pharmacy and helped identify barriers to communicating with the community pharmacist. The reliability of the overall scale in the current study was confirmed, with a Cronbach alpha = 0.945; patients’ expectation index had a Cronbach alpha of 0.773, while the perceived barriers to communication had a Cronbach alpha of 0.888.

#### The patient perception index

This measure was used to determine the perception of patients on the role of the community pharmacist (Iskandar et al., 2017). A higher score indicates a better patient perception of the pharmacist’s role. In this study, the reliability analysis yielded a Cronbach alpha of 0.899.

#### The Lebanese medication adherence scale (LMAS-14)

Medication adherence was assessed using the Lebanese Medication Adherence Scale (LMAS-14), a 14-item tool validated in various populations of Lebanese patients (Bou Serhal et al., 2018; Hallit et al., 2021; Ibrahim et al., 2021; Sakr et al., 2022a). Items are divided into three domains related to psychological, forgetfulness, and economic factors and graded on a 4-point scale ranging from ‘always’ to ‘never.’ The total score was calculated by summing all items, with higher values denoting worse medication adherence. The reliability and sampling adequacy metrics were excellent in this study (Cronbach alpha = 0.927; Barlett's test of sphericity *P* < 0.001; Kaiser-Meyer-Olkin (KMO) = 0.932).

#### The 5-level euroqol (EQ-5D-5L)

The EQ-5D-5L, developed by the EuroQol Group and comprising the EQ-5D descriptive system and the EQ visual analog scale (EQ-VAS), was used to assess patient overall health and well-being (EuroQol Research Foundation, 2021). Respondents indicate their health state in each of the five dimensions covered by EQ-5D (mobility, self-care, usual activities, pain/discomfort, and anxiety/depression) by choosing the most appropriate option, ranging from no problems (1) to slight (2), moderate (3), severe (4), and extreme (5) problems. In this study, its Cronbach alpha reliability measure was 0.753. In addition, EQ VAS measures health status ranging from 0 (worst imaginable health) to 100 (best imaginable health). In this study, Cronbach alpha = 0.753.

### Statistical analysis

The SPSS software version 25 was used to analyse data. A descriptive analysis was performed, where categorical variables were expressed as absolute frequencies and percentages and continuous variables as means and standard deviations.

Factor analysis was employed to extract a large number of sociodemographic and health variables into more manageable clusters, referred to as ‘factors.’ Two distinct factors emerged from this process. Factor 1 encompasses sociodemographic factors related to social and financial aspects, including gender, monthly income, work status, and the IFDFW scale. This factor points to social characteristics such as being male, employed, with a higher monthly income, and possessing a more favourable financial status. Factor 2 encompasses health variables and related factors, including health status, chronic diseases, age, and education. This factor indicates an association of worse health status with more chronic diseases, older age, and lower educational attainment.

**Figure 1.**
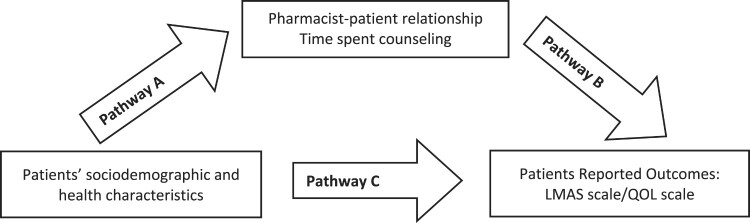
Mediation framework – assessment of the mediation of pharmacists-patient dynamics on the association between patients’ characteristics and reported outcomes.

The PROCESS SPSS Macro version 3.4 model four was used to calculate three pathways in the mediation analysis. While pathway A determined the regression coefficient for the effect of sociodemographic factors on the pharmacist-patient relationship and time spent counseling, pathway B examined the association between the pharmacist-patient relationship and time spent counseling on the LMAS scale and QOL and pathway C estimated the total and direct effect of sociodemographic factors on the LMAS scale and QOL (Figure 1). The Macro generated bias-corrected bootstrapped 95% confidence intervals (CI) to test the significance of the indirect effect. Mediation was deemed significant when the CI around the indirect effect did not include zero. A *p*-value less than 0.05 was considered statistically significant.

## Results

### Description of the sample

In all, 865 respondents participated in this study. The mean age was 32.52 ± 14.56 years, and 39.9% were married. The majority of participants (68.8%) were female, unemployed (51.1%), and had completed university education (78.3%). Regarding their health, 79.3% reported having no chronic disease, and 77.6% stated they had easy access to healthcare; meanwhile, 57.7% indicated that getting nonprescription medications, such as analgesics, anti-inflammatories, or supplements, was the main reason for visiting a community pharmacy. The mean current family size was 4.78 ± 2.40, and the mean financial well-being score was 39.88 ± 18.11. The average routine intake of medications per day was 0.87 ± 1.78. Further descriptive details are available in other articles (Hajj et al., 2023; Sacre et al., 2023b).

### Mediation analysis summary

Table 1 presents the correlation between sociodemographic factors and treatment adherence (LMAS scale) and quality of life (EQ-5D), with mediation analysis involving the pharmacist-patient relationship and counseling time.

**Table 1.** Mediation analyses.

Analysis 1 taking sociodemographic factors as the independent variables, the pharmacist-patient relationship and time spent counseling as mediators, and the LMAS scale as the dependent variable.						
	**Direct effect**			**Indirect effect**		
	**Beta**	**SE**	***p-value***	**Beta**	**Boot SE**	**Boot CI**
**Sociodemographic factors (gender, monthly income, work status, IFDFW scale) on the LMAS scale mediated by pharmacist-patient relationship scales and time spent counseling**						
Patient Expectation Index	0.61	0.38	0.113	0.04	0.04	−0.01; 0.13
Barriers to communicating with the community pharmacist	0.65	0.38	0.086	0.0005	0.01	−0.03; 0.03
Patient Perception Index	0.64	0.38	0.090	0.007	0.03	−0.04; 0.07
Time spent counseling	0.72	0.39	0.067	0.004	0.02	−0.04; 0.05
**Health characteristics (age, health status, chronic disease, education) on the LMAS scale mediated by pharmacist-patient relationship scales and time spent counseling**						
Patient Expectation Index	0.77	0.38	0.042	−0.02	0.02	−0.08; 0.02
Barriers to communicating with the community pharmacist	0.75	0.38	0.048	0.001	0.01	−0.03; 0.03
Patient Perception Index	0.76	0.38	0.046	−0.005	0.02	−0.05; 0.03
Time spent counseling	0.78	0.39	0.046	0.019	0.02	−0.02; 0.08
**Analysis 2 taking sociodemographic factors as the independent variables, the pharmacist-patient relationship and time spent counseling as the mediators, and the QOL scale as the dependent variable.**						
	**Direct effect**			**Indirect effect**		
	**Beta**	**SE**	***p-value***	**Beta**	**Boot SE**	**Boot CI**
**Sociodemographic factors (gender, monthly income, work status, IFDFW scale) on the QOL scale mediated by pharmacist-patient relationship scales and time spent counseling**						
Patient Expectation Index	5.61	0.74	<0.001	0.44	0.22	0.04; 0.95*
Barriers to communicating with the community pharmacist	6.05	0.76	<0.001	0.0002	0.02	−0.05; 0.05
Patient Perception Index	5.83	0.75	<0.001	0.22	0.14	−0.03; 0.54
Time spent counseling	6.27	0.78	<0.001	−0.01	0.08	−0.19; 0.14
**Health characteristics (age, health status, chronic disease, education) on the QOL scale mediated by pharmacist-patient relationship scales and time spent counseling**						
Patient Expectation Index	−6.73	0.72	<0.001	−0.18	0.20	−0.63; 0.17
Barriers to communicating with the community pharmacist	−6.92	0.75	<0.001	0.004	0.02	−0.05; 0.07
Patient Perception Index	−6.80	0.74	<0.001	−0.11	0.14	−0.43; 0.17
Time spent counseling	−6.79	0.78	<0.001	−0.07	0.08	−0.27; 0.06

* Significant mediation. LMAS = Lebanese Medication Adherence Scale. QOL = Quality of Life

The direct and indirect effects of the association between sociodemographic factors and the LMAS scale did not reach significance, indicating no mediation effect. For health characteristics, while the direct effect on the LMAS scale was significant, the absence of a significant indirect effect suggests no mediation.

Regarding the association of sociodemographic factors with quality of life, the direct effect was significant across all mediation models. Notably, the indirect effect was only significant when considering the patient expectation index as a mediator, implying partial mediation. Regarding health characteristics and quality of life, while the direct effect was significant, no significant indirect effect was observed, indicating no mediation.

### Mediation analysis details

**Figure 2.**
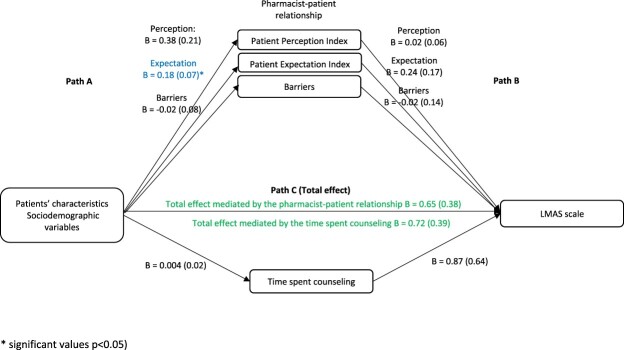
The association between sociodemographic characteristics and the LMAS scale: assessment of the mediation by the pharmacist-patient relationship and time spent counseling. Values are presented as Beta (SE).

In the first analysis, taking the sociodemographic factors as the independent variables and the LMAS scale as the dependent variable, the results showed that in the first step (Path A), the effect of sociodemographic factors on the pharmacist-patient relationship and time spent counseling was not significant, except for the association between sociodemographic factors and expectation (Beta = 0.18, *p* = 0.014). In the second step (Path B), the effect of the pharmacist-patient relationship and time spent counseling on the LMAS scale was not significant. In the third step (Path C (total effect)), the effect of sociodemographic factors on the LMAS scale was not significant (Figure 2).

**Figure 3.**
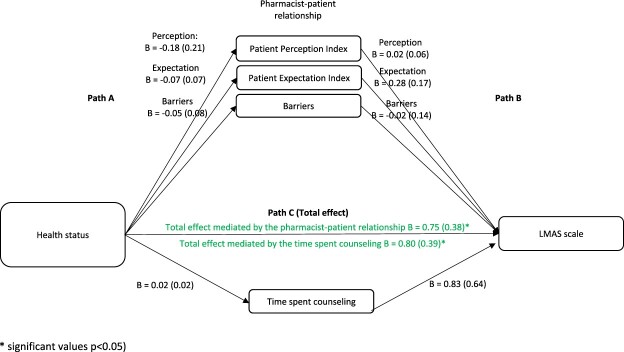
The association between health status and the LMAS scale: assessment of the mediation by the pharmacist-patient relationship and time spent counseling. Values are presented as Beta (SE).

When considering health characteristics as the independent variables and the LMAS scale as the dependent variable, the results showed that in the first step (Path A), the effect of health characteristics on the pharmacist-patient relationship and time spent counseling was not significant (*p* > 0.05 for all). In the second step (Path B), the effect of the pharmacist-patient relationship and time spent counseling on the LMAS scale was not significant. In the third step (Path C (total effect)), the effect of health characteristics on the LMAS scale was significant (Figure 3).

**Figure 4.**
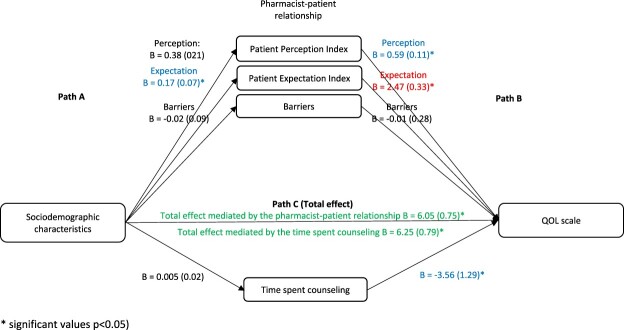
The association between sociodemographic characteristics and QOL: assessment of the mediation by the pharmacist-patient relationship and time spent counseling. Values are presented as Beta (SE).

Taking sociodemographic factors as the independent variables and QOL as the dependent variable, the results showed that in the first step (Path A), the effect of sociodemographic factors on the pharmacist-patient relationship and time spent counseling was not significant, except for the association between demographic factors and expectation (Beta = 0.17, *p* = 0.016). In the second step (Path B), the effect of the pharmacist-patient relationship on QOL was significant for the expectation (Beta = 2.47, *p* < 0.001), perception (Beta = 0.59, *p* < 0.001), and time spent counseling (Beta = −3.56, *p* = 0.006). In the third step (Path C (total effect)), the effect of sociodemographic factors on QOL was significant (Figure 4).

**Figure 5.**
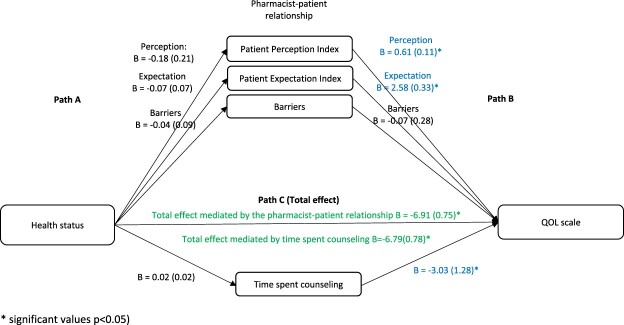
The association between health variables and QOL: assessment of the mediation by the pharmacist-patient relationship and time spent counseling. Values are presented as Beta (SE).

When considering health characteristics as the independent variables and QOL as the dependent variable, the results showed that in the first step (Path A), the effect of health characteristics on the pharmacist-patient relationship and time spent counseling was not significant (*p* > 0.05 for all). In the second step (Path B), the effect of the pharmacist-patient relationship on QOL was significant for the expectation (Beta = 2.58, *p* < 0.001), perception (Beta = 0.61, *p* < 0.001), and time spent counseling (Beta = −3.03, *p* = 0.018). In the third step (Path C (total effect)), the effect of health characteristics on QOL was significant (Figure 5).

## Discussion

This study investigated the intricate interplay between sociodemographic factors, treatment adherence, and quality of life (QOL), with a particular focus on the mediating role of the pharmacist-patient relationship and counseling time. Our key findings reveal a compelling association between worse health status and both increased medication non-adherence, as measured by the LMAS scale, and reduced QOL. Furthermore, sociodemographic factors were found to be correlated with QOL, highlighting the improved well-being among men with higher income and better financial wellness.

Despite the considerable impact of demographic factors on patient expectations, our study challenges the expected mediation role of the pharmacist-patient relationship and counseling time on medication adherence. Nevertheless, patient expectations partially mediated the relationship between sociodemographic characteristics and quality of life (QOL). Our last finding underscores the positive influence of the pharmacist-patient relationship on QOL, with higher expectations and favourable perceptions associated with improved well-being.

Consistent with previous findings (Chantzaras & Yfantopoulos, 2022; Ho et al., 2009; Piña et al., 2021; Wu & Moser, 2021), worse health status was associated with worse adherence (higher LMAS scores) and worse QOL, although LMAS and QOL were not significantly related (results not shown). In the present study, most participants were ambulatory patients with no chronic diseases and visited the pharmacy for acute ailments, which are unlikely to affect QOL (Zhang et al., 2023). Moreover, some studies have found that medication adherence can improve health outcomes, particularly in patients with poor health status (Aremu et al., 2022; Lin et al., 2020), suggesting that further research is needed to elucidate the complex relationship between health status, medication adherence, and quality of life.

While demographic factors seemed to significantly shape patient expectations, the absence of mediation through the pharmacist-patient relationship or counseling time on adherence challenges previous results (Garjani et al., 2009; Gebhart, 2019; Oh et al., 2002; Rajiah et al., 2021) and raises questions about the nature of patient expectations and the need for further exploration in diverse populations. Although the patient-pharmacist relationship and time spent counseling (more than 10 min) have been found to independently affect adherence (Sakr et al., 2023b), these elements were not enough to counteract the effect of patients’ characteristics on medication adherence, as shown in other studies (Svarstad et al., 2022). Several reasons might underlie this finding. Firstly, the time allocated for counseling may be insufficient and could stem from economic challenges faced by pharmacists (Hallit et al., 2017), leading to difficulties in hiring additional staff and subsequently limiting the available time for patient interactions. This financial constraint may affect the depth and effectiveness of counseling interventions. Secondly, participant dissatisfaction with the counseling received is another conceivable factor, with the quality or relevance of the counseling provided falling short of meeting patient expectations, explained by several factors such as pharmacists’ education and patients’ age, marital status, and literacy (Showande & Laniyan, 2022). This finding highlights a critical aspect that requires attention for optimising pharmacist-patient interactions and ensuring the effectiveness of adherence-related interventions. Other researchers could demonstrate the complex nature of the intervention among diabetes patients, where mediation was absent for some aspects of the pharmacist intervention (improvement of self-efficacy) but present for others (increased satisfaction with medication information) (Hofer et al., 2017).

The significant correlation between sociodemographic factors and QOL is consistent with previous studies that have shown the impact of social and economic factors on the well-being of patients (Braveman & Gottlieb, 2014; Khayyat et al., 2019). Interestingly, patient expectations partially mediated the relationship between sociodemographic characteristics and QOL. This partial mediation effect suggests a potential role for pharmacists in improving QOL across the general population, particularly among vulnerable demographic groups. This finding suggests that, through their interventions, pharmacists can play a mitigating role, partially offsetting the impact of factors such as gender and socioeconomic difficulties on individuals’ quality of life. It also holds significant importance within the Lebanese context, as the population grapples with the repercussions of a severe socioeconomic crisis stemming from various root causes (Sacre et al., 2022; Salameh et al., 2020).

This positive and significant mediating effect of the pharmacist-patient relationship on QOL is consistent with previous conclusions showing the importance of this relationship on patients (Schulz et al., 2020; Syarifuddin et al., 2019; Kelly et al., 2023). In addition, patient perception of the pharmacist was independently linked to a better QOL, while barriers to communications, as previously reported, did not show a similar association (Hajj et al., 2023). However, the unexpected negative correlation between longer counseling times and lower quality of life diverges from conventional expectations (Cahoon, 2023; Fajriansyah et al., 2020). One possible explanation could be reverse causality, as patients with worse QOL need lengthier counseling and listening sessions. This result suggests a move away from a one-size-fits-all approach to counseling duration and highlights the need for tailored interactions that consider patient preferences and information-processing abilities and warrant further investigation.

Lastly, the findings from this study underscore the need to revisit and update the existing pharmaceutical policy framework in Lebanon. Currently, pharmacy practice in Lebanon is governed by Law No. 367 of August 1, 1994. This law does not address modern pharmacy concepts such as patient counseling, medication therapy management, or other clinical services. Additionally, it prohibits pharmacists from performing certain healthcare services, including vaccinations and invasive procedures like injections. This regulatory environment can prevent pharmacists from fulfilling their role as medication experts and patient advocates, limiting their ability to provide effective counseling and build strong relationships with patients. A comprehensive review and update of this policy, informed by the present findings and contemporary pharmacy practices, could enable pharmacists to have a more significant impact on medication adherence and quality of life outcomes.

This study contributes to a more comprehensive understanding of the complex interplay between patient characteristics, pharmacist-patient dynamics, and patient outcomes. While the pharmacist-patient relationship independently influences medication adherence and quality of life, communication with the patient does not appear to be sufficient to mitigate the impact of health and socioeconomic difficulties, except for patient expectations regarding quality of life. Further research is warranted to delve into the underlying mechanisms and contextual factors that affect these relationships, allowing for the refinement and personalisation of pharmaceutical care strategies.

### Implications for clinical practice and healthcare policy

- Pharmacists should adopt a personalised approach to patient counseling, involving more in-depth and targeted discussions for patients with complex health and medication regimens.
- Pharmacists should build strong, trust-based relationships with patients from diverse socioeconomic and cultural backgrounds by improving their communication skills and understanding patient expectations to positively influence their quality of life.
- Pharmacists should enhance their ability to deliver effective counseling and support by engaging in continuing education and professional development programmes, particularly focusing on managing chronic diseases and understanding the socioeconomic factors affecting patients.
- Clinical decision support systems and medication adherence monitoring tools could be implemented in pharmacies to help pharmacists identify patients at risk of non-adherence and providing targeted interventions.
- Policymakers should consider revising and updating the existing pharmacy laws in Lebanon to reflect modern pharmacy practices and enable pharmacists to provide a broader range of services, including medication therapy management, point-of-care testing, and patient counseling, among others.
- Policymakers should implement reimbursement measures that address the economic challenges faced by pharmacists, such as financial incentives or subsidies for hiring additional staff, to compensate pharmacists for providing comprehensive counseling and adherence support services.
- Policymakers should promote interprofessional collaboration and encourage the integration of pharmacists into public health initiatives as contributors to health promotion and disease prevention programmes, especially in underserved and vulnerable populations.
- Policymakers should raise public awareness through campaigns and educational initiatives to educate patients on the expanding role of pharmacists and the benefits of medication adherence.

### Directions for future research

- Conduct longitudinal, interventional studies to explore the long-term effects of pharmacist-led interventions on patient satisfaction, counseling effectiveness, medication adherence, and health outcomes, providing more comprehensive insights into these aspects over time. A targeted intervention, the IMPHACT-LB Project Phase 2, is currently ongoing; it aims to assess patient satisfaction, adherence, and overall well-being pre- and post-pharmacist intervention.
- Investigate the complex relationship between socioeconomic factors, medication adherence, and QOL, focusing on specific barriers and facilitators to effective pharmaceutical care in different socioeconomic contexts.
- Explore the effectiveness of different approaches and tools, including telepharmacy, shared decision-making aids, and mobile health applications, in improving medication adherence and quality of life, particularly in underserved and remote areas.
- Conduct qualitative studies for deeper insights into patients’ perspectives, experiences, and preferences regarding their interactions with pharmacists and counseling services.
- Conduct studies to evaluate the effectiveness of interprofessional collaboration involving pharmacists, physicians, and other healthcare professionals in improving medication adherence and patient-centered care.
- Develop and validate assessment tools to measure the quality of pharmacist-patient interactions and their impact on patient outcomes.

### Study limitations

While this work breaks new ground by exploring the mediation effect of pharmacist-patient dynamics on patients’ medication adherence and quality of life, it is not without limitations. The study design precludes establishing causality, and the method employed does not eliminate the possibility of selection bias or information bias, both of which may result in an underestimation of the findings. However, the study holds strengths such as an adequate sample size, rigorous standardised analysis, and the meticulous consideration of social, economic, educational, and health characteristics in the complex models employed. Future research of a prospective or interventional nature is warranted to validate our findings. Exploring the moderating effect of pharmacist-patient dynamics could further enrich our understanding of this concept.

## Conclusion

This study sheds light on the intricate dynamics between patient characteristics, health status, medication adherence, and quality of life within the context of the patient-pharmacist relationships. The findings underscore the multifaceted influences on medication adherence and quality of life, emphasising the significance of patient expectations and the pharmacist-patient relationship. While uncovering unexpected nuances, such as the negative association between longer counselling times and lower quality of life, this research points to the need for tailored interventions. Future studies could delve deeper into understanding the specific factors driving patient expectations, refine counselling strategies, and explore innovative approaches to enhance the pharmacist's role in improving patient outcomes. These insights pave the way for targeted interventions, which could improve medication adherence and overall well-being among diverse patient populations.

## Data Availability

The datasets generated during and/or analysed during the current study are available from the corresponding author on reasonable request.

## List of abbreviations

IMPHACT-LB: Impact of the Modern Pharmacy Concept on Patient Therapy in Lebanon, QOL: quality of life, IFDFW: InCharge Financial Distress/Financial, KMO: Kaiser-Meyer-Olkin, LMAS: Lebanese Medication Adherence Scale, KMO: Kaiser-Meyer-Olkin, EQ-5D-5L: EuroQol five-dimensional five level, EQ-VAS: EuroQol visual analog scale, SPSS: Statistical package of social science, SE: Standard error, CI: confidence level.
